# Advanced glycation endproducts mediate chronic kidney injury with characteristic patterns in different stages

**DOI:** 10.3389/fphys.2022.977247

**Published:** 2022-09-09

**Authors:** Xiaoxia Huang, Bingyu Li, Jiaqing Hu, Zhuanhua Liu, Dongping Li, Zhenfeng Chen, Hang Huang, Yanjia Chen, Xiaohua Guo, Yun Cui, Qiaobing Huang

**Affiliations:** ^1^ Guangdong Provincial Key Laboratory of Cardiac Function and Microcirculation, Department of Pathophysiology, School of Basic Medical Sciences, Southern Medical University, Guangzhou, China; ^2^ The Second School of Clinical Medicine, Southern Medical University, Guangzhou, China; ^3^ Department of Anesthesiology, Nanfang Hospital, Southern Medical University, Guangzhou, China; ^4^ Shunde Hospital, Southern Medical University (The First People’s Hospital of Shunde), Guangzhou, China

**Keywords:** advanced glycation endproducts, RAGE, angiogenesis, renal fibrosis, moesin phosphorylation

## Abstract

Advanced glycation endproducts (AGEs) have been confirmed to play a causative role in the development of diabetic nephropathy (DN). In this study, we revealed that AGE-induced kidney injury with characteristic patterns in different stages and moesin phosphorylation plays a role in these processes. In WT mice treated with AGE-modified bovine serum albumin (AGE-BSA), distinct abnormal angiogenesis in Bowman’s capsule of the kidney emerged early after 1 m under AGE-BSA stimulation, while these neovessels became rare after 6 m. AGE-BSA also induced glomerular hypertrophy and mesangial expansion at 1 m but glomerular atrophy and fibrosis at 6 m. Electron microscopy imaging demonstrated the damage of foot process integrity in podocytes and the uneven thickening of the glomerular basement membrane in the AGE-BSA-treated group, which was more significant after 6 m of AGE-BSA treatment than 1 m. The kidney dysfunction appeared along with these AGE-induced morphological changes. However, these AGE-BSA-induced pathological changes were significantly attenuated in RAGE-knockout mice. Moreover, moesin phosphorylation was accompanied by AGE-BSA-induced alterations and moesin deficiency in mice attenuated by AGE-BSA-induced fibrosis. The investigation on glomerular endothelial cells (GECs) also confirmed that the phosphorylation of moesin T558 is critical in AGE-induced tube formation. Overall, this study suggests that AGEs mediate kidney injury with characteristic patterns by binding with RAGE and inducing moesin phosphorylation.

## Introduction

Diabetic nephropathy (DN), one of the most common complications of diabetes mellitus, is a progressive kidney disease with high morbidity and mortality. The development and progression of DN is initiated by hyperglycemia. It is also believed that advanced glycation endproducts (AGEs) play an important role in diabetic complications (Yamagishi et al., 2015; Brings et al., 2017; Lee et al., 2018). AGEs, the non-enzymatically glycosylated protein derivatives formed during prolonged hyperglycemia exposure, have been proven to accumulate in plasma and tissues not only in patients with diabetes (Thomas et al., 2015) but also in patients with metabolic dysfunction and aging due to the insufficient scavenging (Chaudhuri et al., 2018). It is reported that the kidney is the main organ of AGE deposit in diabetic patients (Kumar Pasupulati et al., 2016; Rabbani and Thornalley, 2018).

DN is also regarded as a microvascular complication of diabetes and the impairment of endothelial cells, and the subsequent microvascular dysfunction plays an important role in the development of DN (Cheng et al., 2022; Kanno et al., 2022). Our previous studies have proved that AGEs induce excessive angiogenesis in human umbilical vein endothelial cells (HUVECs) and AGE-treated mice (Wang et al., 2016; Chen et al., 2020). Whether AGEs also induce renal angiogenesis in AGE-mediated glomerular injury has not been investigated.

Experiments of *in vivo* and *in vitro* studies suggested that the interaction between AGEs and their receptor (RAGE) elicited severe effects on renal failure through multiple intracellular signaling pathways, and the inhibition of AGEs or RAGE has become a potential target for DN management (Yamamoto et al., 2001; Yiu et al., 2016; Kim et al., 2018; Azegami et al., 2021).

The ERM proteins (ezrin, radixin, and moesin) are members of the band 4.1 superfamily. They function as a linker between transmembrane proteins and cytoskeleton, participating in cell polarity, mitosis, migratory behavior, lumen morphogenesis, and cell signaling (Jankovics et al., 2002; Polesello et al., 2002; Göbel et al., 2004; Kunda et al., 2008; Fehon et al., 2010). Studies have shown that ERMs are activated when they adopt an open structure, following phosphorylation of the regulatory threonine T576, T564, and T558 in ezrin, radixin, and moesin, respectively. Moesin is the major ERM protein expressed in endothelial cells, and the phosphorylation of moesin on T558 residue is involved in the AGE-induced angiogenic process (Wang et al., 2016). Our studies also indicated that AGEs induced moesin phosphorylation both in the brain and retina (Li et al., 2011; Wang et al., 2012; Chen et al., 2020). But it is still not clear whether moesin phosphorylation participates in AGE-induced renal endothelial dysfunction and angiogenesis.

In the present study, we established an AGE-BSA-stimulated animal model in C57BL/6 wild-type (WT), RAGE-knockout, and moesin-knockout mice to elicit AGE-induced kidney injury. AGE/RAGE-induced renal angiogenesis and fibrosis were compared in different time periods. The role of moesin phosphorylation in this process was also investigated.

## Material and methods

The study was approved by the Institutional Review Board of Southern Medical University.

### Antibodies and reagents

Reagents were purchased from Sigma-Aldrich (St. Louis, MO, United States), unless otherwise indicated. Antibodies against phospho-moesin (T558), moesin, and CD34 were purchased from Abcam (Cambridge, UK). β-actin antibody was obtained from Cell Signaling Technology (Beverly, MA, United States). Biotin-free horseradish peroxidase (HRP)-conjugated secondary antibodies were purchased from Sigma (St. Louis, MO, United States). Tyramide-CY3 and FITC were obtained from Servicebio (Wuhan, China). DAPI was obtained from BestBio (Shanghai, China). RIPA Lysis Buffer, PMSF, phosphatase inhibitor cocktail, and bovine serum albumin (BSA) were all obtained from GBCBIO (Guangzhou, China). Hematoxylin and Eosin (HE) Stain Kit, Periodic Acid Schiff (PAS) Stain Kit, and Masson’s trichrome Stain Kit were purchased from Solarbio (Beijing, China).

### Preparation of advanced glycation endproduct–bovine serum albumin

BSA-modified AGEs (AGE-BSA) were prepared, as previously reported by Wang et al., (2016). Briefly, BSA (150 mmol/L, pH 7.4) was incubated in PBS with D-glucose (250 mmol/L) at 37°C for 8 weeks, while the control albumin was incubated without glucose. At the end of the incubation period, both solutions were extensively dialyzed against PBS and purified. The endotoxin content was detected by Limulus amoebocyte lysate assay (Sigma, St. Louis, MO, United States) and was found to be less than 500 U/L in both solutions. AGE-specific fluorescence was determined by ratio spectrofluorometry, showing that AGE-BSA contained an AGE content of 74.802 U/mg protein, while native albumin had an AGE content of less than 0.9 U/mg protein.

### Animal models

Male C57BL/6 wild-type (WT) mice were purchased from the Laboratory Animal Center of Southern Medical University. RAGE-knockout mice (RAGE ^(−/−)^) were kindly provided by Kanazawa University, Japan (Myint et al., 2006; Chen et al., 2020). Moesin-knockout mice (Msn^−/Y^) were constructed through CRISPR/Cas-mediated genome engineering by a commercial supplier (Cyagen). Briefly, exon 4 to exon 8 were selected as target sites. Cas9 mRNA and gRNA generated by *in vitro* transcription were then co-injected into fertilized eggs for knockout mouse production (Doi et al., 1999; Ansa-Addo et al., 2017). All mice had free access to standard mouse chow and tap water. All animals were treated according to protocols approved by the Animal Care and Ethical Committee of Southern Medical University. Mice aged 6–8 weeks were given an intraperitoneal injection with AGE-BSA (10 mg per kg per day) or with saline and native BSA as comparisons for 1 month or 6 months. On the last day of the stimulation period, mice were anesthetized by intraperitoneal injection of 2% sodium pentobarbital (30 mg/kg) for the harvest of blood and urine samples and kidney tissues.

### Immunohistochemical staining

Paraffin-embedded mouse kidney sections (3 μm thickness) were allowed to go through deparaffinization and antigen retrieval. Then, primary antibodies against CD34 or phospho-moesin (T558) were, respectively, applied overnight at 4°C. Next, slides were washed three times and incubated with the HRP-labeled antibody as the secondary antibody. Subsequently, the HRP complex was identified with 3, 3′-diaminobenzidine (DAB) with the DAB substrate working solution, followed by dehydration. Finally, sections with coverslips were mounted and photographed using a microscope (Zeiss Imager Z2, Germany). Angiogenetic vessels were evaluated by the number of lumen-like CD34-positive areas around glomeruli. The phospho-moesin-positive area was measured by ImageJ. All measurements were performed in a blind manner.

### Transmission electron microscopy

Briefly, kidneys were removed from mice and immediately fixed in 2.5% glutaraldehyde. Then, the kidney cortex was carefully cut into pieces of 1 mm × 1 mm × 1 mm and fixed at room temperature for 1 h and 4°C overnight. Next, samples were rinsed with PBS several times, followed by dehydration in a graded series of acetone. After that, samples were permeated and embedded in epoxy resin for trimming and ultrathin sectioning. Ultrathin slices were cut to 90 nm thickness and were stained with lead citrate and uranyl acetate. Finally, images were visualized by transmission electron microscopy (TEM) (Hitachi H-7500, Japan). The integrity of foot processes in podocytes was assessed by counting the number of foot process junctions per micron of the basement membrane by ImageJ (Verma et al., 2018). Kidneys from at least four animals were analyzed in each experimental condition.

### Histological and morphometric assessments

The same region of the kidney in mice was obtained to conduct the histological assay. Briefly, kidney tissues were fixed in 4% paraformaldehyde at room temperature and embedded in paraffin according to a standard protocol. Paraffin-embedded mouse kidney sections (3 μm thickness) were stained with HE, PAS, or Masson trichrome reagent, following manufacturer’s protocols for renal morphological observation and morphometric assessment. Sections were observed using a light microscope (Zeiss Imager Z2, Germany). The glomerular areas in HE staining, PAS, and Masson’s trichrome staining-positive area were all analyzed by ImageJ in a blinded manner. As PAS staining indicates the mesangial matrix and basement membrane and Masson’s staining indicates the deposit of collagen fibrils, renal fibrosis was presented by the ratio of PAS or Masson-positive area/tissue area.

### Analysis of biochemical indexes in mouse urine and serum

Mice were treated with AGE-BSA for 1 m or 6 m. At the end of treatment, the urine of mice was collected by bladder irritation, and blood was obtained through the eyeball method under anesthesia. After being kept on ice for 30 min, blood was centrifuged at 3,000 rpm for 10 min to make the serum segregated. Both urine and serum were stored at −80°C. Samples were detected by using an Automatic Biochemical Analyzer (Chemray 240; Rayto) to determine the levels of urine creatinine and albumin, blood creatinine, and blood urea nitrogen. The level of albuminuria was presented as the ratio of urine albumin/urine creatinine.

### Western blot analysis

The tissue protein of the kidney cortex section was extracted by RIPA Lysis Buffer containing the proteinase inhibitor (1 mM PMSF and 1 mM phosphatase inhibitor cocktail). The protein concentrations were measured by using a BCA Protein Assay Kit (Cat. G3522-1; GBCBIO). Equal amounts of protein were subjected to SDS-PAGE electrophoresis and transferred to polyvinylidene fluoride (PVDF) membranes. After being blocked with 5% BSA in TBS containing 0.1% Tween-20 (TBS-T) for 1 h, the membranes were incubated with the anti-phospho-moesin (T558) antibody (1:1000) and anti-moesin antibody (1:1000) at 4°C overnight, followed by incubating with homogenous HRP-conjugated secondary antibodies at room temperature for 1 h. Finally, protein bands were visualized by chemiluminescence. Band density was analyzed by ImageJ.

### Detection of phospho-moesin and CD34 localization with the confocal microscope

As the anti-phospho T558 moesin and anti-CD34 antibodies we used were homologous, TSA (tyramide signal amplification) technology was applied for renal double immunofluorescence staining (phosphor-moesin and CD34), as reported by Zhang et al. (2017). Briefly, tissue sections were punched with 0.3% Triton X-100 and blocked with blocking solution for 30 min at room temperature. Then, sections were incubated with the anti-phospho T558 moesin antibody (1:100) at 4°C overnight. After being washed three times for 5 min each with PBS, sections were incubated with corresponding HRP-conjugated secondary antibodies (1:200) for 1 h at room temperature. Subsequently, tyramide-CY3 was added to the section for 10 min at room temperature, followed by washing three times for 5 min each. After that, microwaving treatment was conducted to wash away the non-covalently bound antibody, while covalently bound fluorescein remained on the sample. Next, sections were proceeded for the incubation of the CD34 antibody (1:100), and after HRP-conjugated secondary antibodies’ incubation, tyramide-FITC was added to the section. Cell nuclei were stained with DAPI (1:50) for 10 min at room temperature. Finally, pictures were taken with a Zeiss LSM780 laser confocal scanning microscope (Zeiss, Germany), and digital images were imported into ImageJ for fluorescence-integrated density measurement. Mander’s overlap colocalization coefficients (mean ± SEM) were generated by Zen software to estimate colocalization. Values of Mander’s overlap colocalization coefficients from 0.6 to 1.0 were considered colocalization, while values from 0 to 0.6 indicated the absence of colocalization (Zinchuk and Grossenbacher-Zinchuk, 2009).

### Cell culture

Glomerular endothelial cells (GECs) were obtained from BeNa Culture Collection (BNCC). DMEM medium with high glucose (DMEM-H) and fetal bovine serum (FBS) were obtained from Gibco. According to the descriptions of the manufacturer, GECs were cultured in DMEM-H with 10% FBS at 37°C in a humidified atmosphere with 5% carbon dioxide. Cells between the fourth and sixth passages were used for the corresponding experiment.

### Adenovirus construction and transfection

The wild-type (WT) moesin overexpressing adenovirus and the moesin mutant adenoviruses T558D (Thr-558→Asp, phospho-constitutive active flag-moesin-T558D) and T558A (Thr-558→Ala, phospho-deficient flag-moesin-T558A) were designed and synthesized by GeneChem (Shanghai, China). The transfection of adenovirus was carried out according to the instruction book provided by the manufacturer with slight modifications. Briefly, GECs were cultured to 30%–50% confluences. Then, adenovirus was added to GECs for 48 h with a multiplicity of infection (MOI) of 50, followed by corresponding experiments. The efficiency of intervention on moesin phosphorylation in GECs was evaluated by Western blotting.

### Tube formation detection of glomerular endothelial cells

The assay was performed by utilizing Matrigel (Corning Inc., Corning, NY, United States). First, 50 μl Matrigel was added to a 96-well plate, and the gel was allowed to solidify at 37°C for 1 h. Subsequently, GECs transfected with or without adenovirus were seeded onto the layer of Matrigel (8 × 10^4^ cells/well), followed by 10 h of incubation with or without AGE-BSA (100 μg/mL) (Wang et al., 2016). At the end of incubation, tubular network structures were visualized and photographed using a phase contrast microscope. The total tube length and the branch points were measured by ImageJ.

### Statistical analyses

Data were analyzed by GraphPad Prism version 8.0. All data were presented as mean ± SD of at least three independent experiments, unless otherwise noted. Statistical comparisons were performed using one-way ANOVA, followed by an appropriate *post hoc* test. *P* < 0.05 was considered significant.

## Results

### Advanced glycation endproducts- induced characteristic angiogenesis in the kidney at an early stage

**FIGURE 1:**
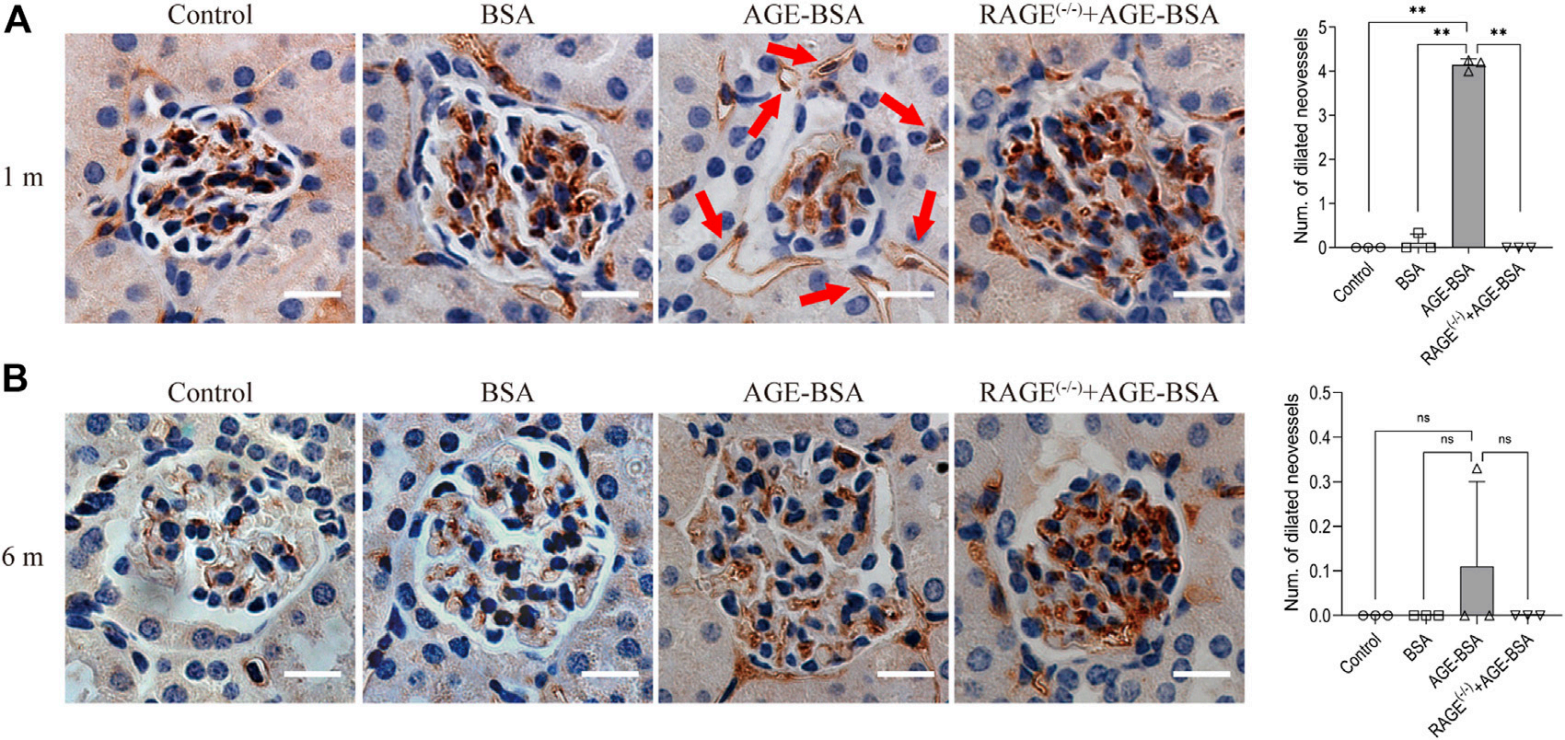
AGE-BSA induces RAGE-mediated renal angiogenesis in the early stage of stimulation. Representative images of CD34 staining of glomeruli after AGE-BSA treatment for 1 m **(A)** and 6 m **(B)** are shown, and the number of dilated vessels around glomeruli was counted and analyzed. Arrow: dilated neovessels around the renal capsule. Scale bar = 20 μm. *n* = 3, **p < 0.01, ns: no significance.

One month after consecutive intraperitoneal injection of AGE-BSA, the staining of endothelial cell-specific CD34 illustrated remarkable dilated neovessels around Bowman’s capsule of glomeruli in the kidney, indicating the development of angiogenesis at the early stage of AGE-BSA stimulation (Figure 1A, arrow). The results at 6 m showed few positive areas of CD34 staining and no more dilated microvessels in the AGE-BSA-treated group (Figure 1B).

**FIGURE 2:**
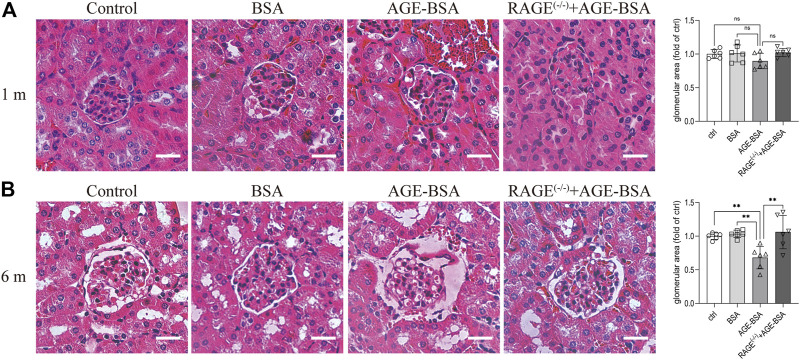
AGE-BSA induces RAGE-mediated renal disorganization with vessel dilation and congestion. HE staining of renal tissue was performed at 1 m **(A)** and 6 m **(B)** after treatment. Scale bar = 40 μm. The glomerular area was quantitatively analyzed by ImageJ (right panels). *n* = 6. **p < 0.01.

Consistent with the results of CD34 staining, HE staining images revealed that there were obvious dilated microvessels filled with red blood cells around glomeruli and renal tubules in kidneys from AGE-BSA-treated mice for 1 m (Figure 2A), indicating the development of angiogenesis and even microaneurysm at an early stage of AGE-BSA stimulation. The dilated and congested microvessels were absent, and there were glomerular and tubular atrophies with the decreased glomerular area (Figure 2B) and even vacuolation degeneration of tubular epithelial cells in kidneys from AGE-BSA-treated mice at 6 m (Figure 2B). These short-term pro-angiogenic and long-term atrophy effects of AGE-BSA were all attenuated in kidneys from RAGE ^(−/−)^ mice (Figures 1, 2), suggesting the critical role of RAGE in AGE-induced pathological responses in the kidney.

### Advanced glycation endproducts/receptor advanced glycation endproducts induced prominent glomerular ultramicrostructure alteration

**FIGURE 3:**
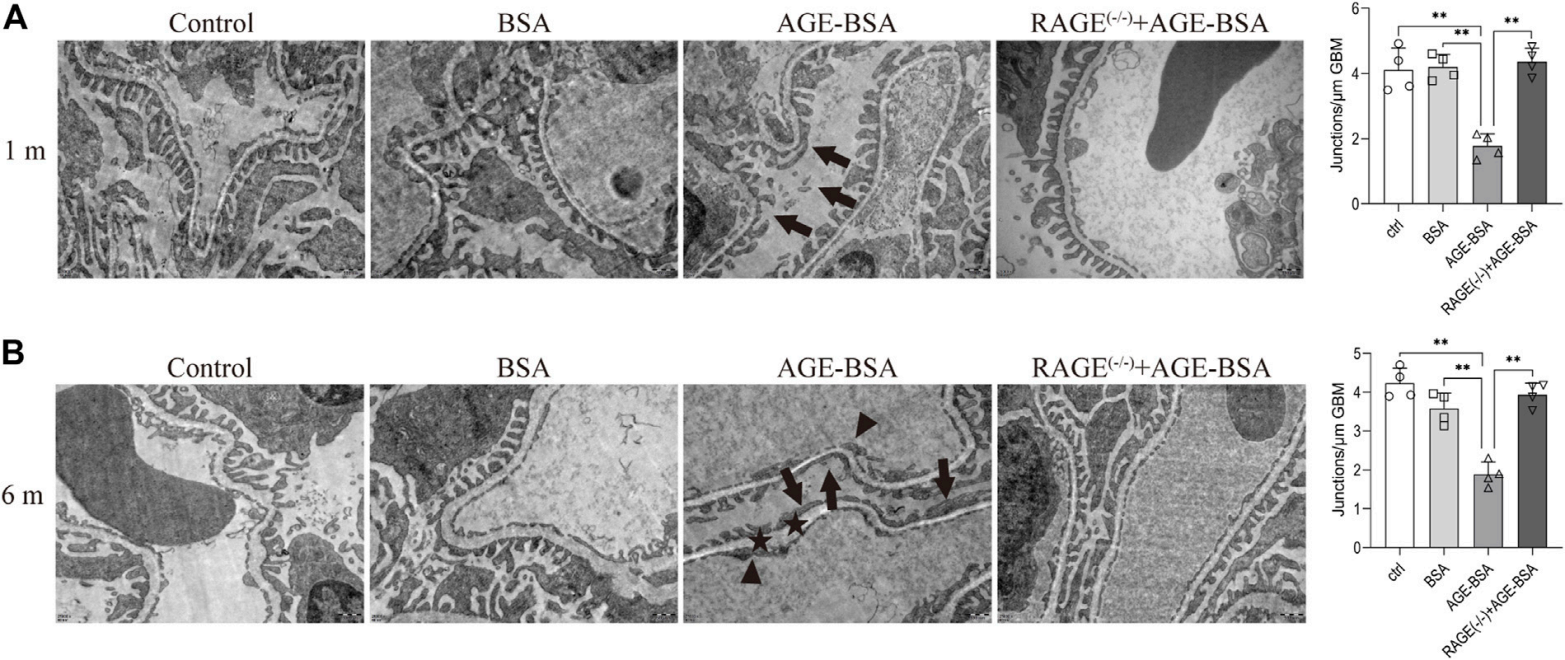
AGE-BSA induces RAGE-mediated ultrastructure alteration of podocytes and basement membrane in renal glomeruli. TEM of renal tissue was performed at 1 m **(A)** and 6 m **(B)** after treatment. The podocyte intercellular junction frequency was quantitatively analyzed (right panels). Arrow: podocyte pseudopodia. Arrowhead: glomerular endothelial cells. Asterisk: basement membrane. Scale bar = 500 nm *n* = 4. *p < 0.05 and **p < 0.01.

TEM was used to determine the ultramicrostructure alterations of the kidney exposed to AGE-BSA. The images demonstrated that there were significant structural changes in podocytes and endothelial cells in AGE-BSA-treated mice. The fusion of foot processes and the detachment of podocytes appeared in kidneys from AGE-BSA-treated mice with the number of foot process junctions decreasing at 1 m (Figure 3A, arrow) and became more obvious at 6 m (Figure 3B, arrow). The lining of endothelial cells in the glomerulus was discontinuing (Figure 3B, arrowhead). There was an expanding and uneven thickness in the basement membrane between podocytes and the endothelium (Figure 3B, asterisk) at 6 m after AGE-BSA stimulation. All these renal damages were ameliorated in kidneys from RAGE^(−/−)^ mice, confirming that AGEs elicit glomerular ultramicrostructure alteration through RAGE.

### Advanced glycation endproduct/receptor advanced glycation endproduct induced significant renal fibrosis at the late stage

**FIGURE 4:**
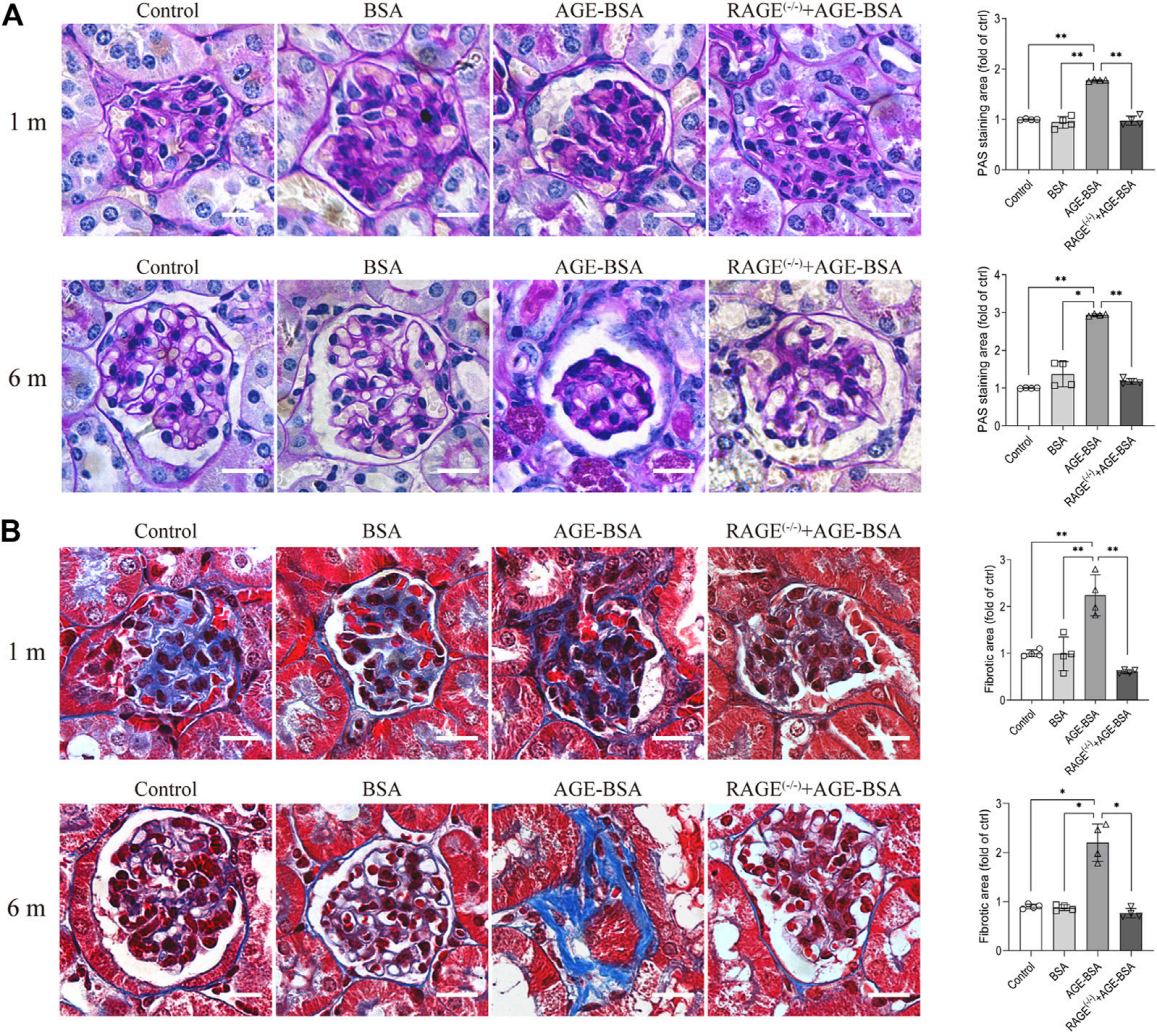
AGE-BSA induces RAGE-mediated renal fibrosis. **(A)** PAS staining and **(B)** Masson’s staining were conducted in renal tissues from WT and RAGE ^(−/−)^ mice after 1 and 6 m AGE treatment. Scale bar = 20 μm. *n* = 4, *p < 0.05, and **p < 0.01.

The results of immunohistochemistry (Figure 1) and TEM (Figure 3) implied AGE-evoked renal angiogenesis at the early stage and glomerular atrophy with glomerular basement membrane (GBM) uneven thickness at the late stage. We further examined the fibrosis of the kidney under AGE-BSA treatment with PAS and Masson’s trichrome staining. Images showed that in WT mice treated with AGE-BSA for 1 m, there was slightly heavier staining of PAS in the glomerulus than that in the control and BSA group (Figure 4A, upper panel). PAS staining was greatly increased in kidneys from AGE-BSA-treated WT mice at 6 m (Figure 4A, lower panel), indicating the thickening of GBM. Histological evaluation of Masson’s trichrome staining exhibited that, compared with the control and BSA group, collagen fibrils were significantly deposited in glomeruli in AGE-BSA-treated WT mice at 1 m (Figure 4B, upper panel). The deposit of collagen was more significant in the interstitial area in the kidney from AGE-BSA-treated WT mice at 6 m with difficulty finding the glomerulus (Figure 4B, lower panel). These results suggest the development of renal fibrosis and confirm that there is glomerular atrophy in long-term AGE challenge. Similarly, no significant changes, either PAS-positive staining or collagen fibril deposition, were observed in AGE-BSA-treated RAGE^(−/−)^ mice, indicating again that AGEs induce renal fibrosis through RAGE *in vivo.*

### Advanced glycation endproduct–bovine serum albumin induced renal dysfunctions

**FIGURE 5:**
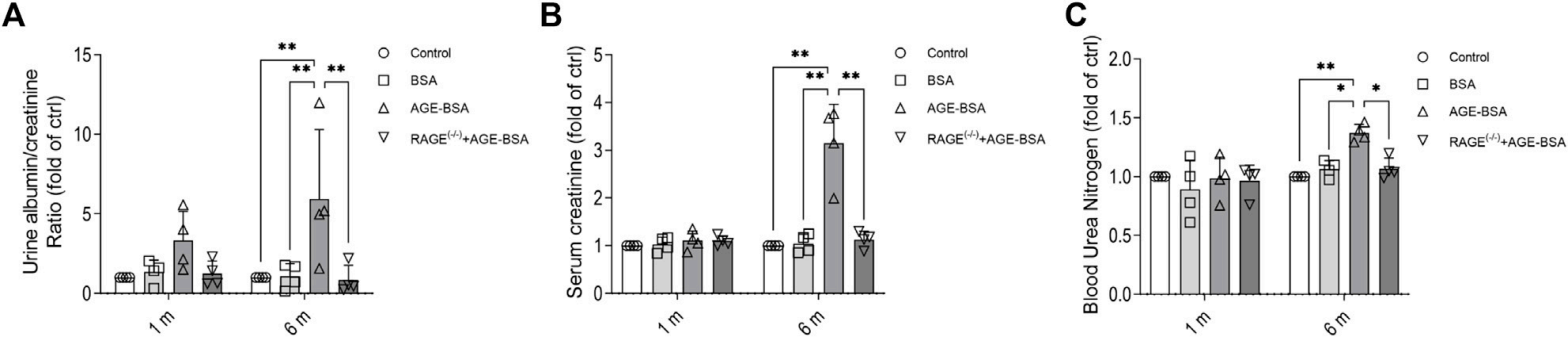
AGE-BSA induces RAGE-mediated renal dysfunction. The ratio of urine albumin to creatinine **(A)** was regarded as the level of albuminuria. Serum creatinine **(B)** and blood urea nitrogen **(C)** were also measured. *n* = 4, *p < 0.05, and **p < 0.01.

The results of renal function assays corresponded with the morphological changes with a significant increase of albuminuria early at 1 m after AGE treatment (Figure 5A), indicating the leakage of albumin from microvessels due to, at least partially, the early pathological angiogenesis and immature neovessels induced by AGE stimulation. The more severe albuminuria (Figure 5A) and the increases of serum CRE (Figure 5B) and BUN (Figure 5C) happened at 6 m after AGE-BSA treatment, suggesting the failure of renal function after the long-term challenge of AGEs. These functional alterations were all inhibited in AGE-BSA-treated RAGE^(−/−)^ mice, suggesting again that RAGE plays an important role in AGE-induced kidney malfunction.

### Phosphorylation of moesin was accompanied by advanced glycation endproduct-induced renal response

**FIGURE 6:**
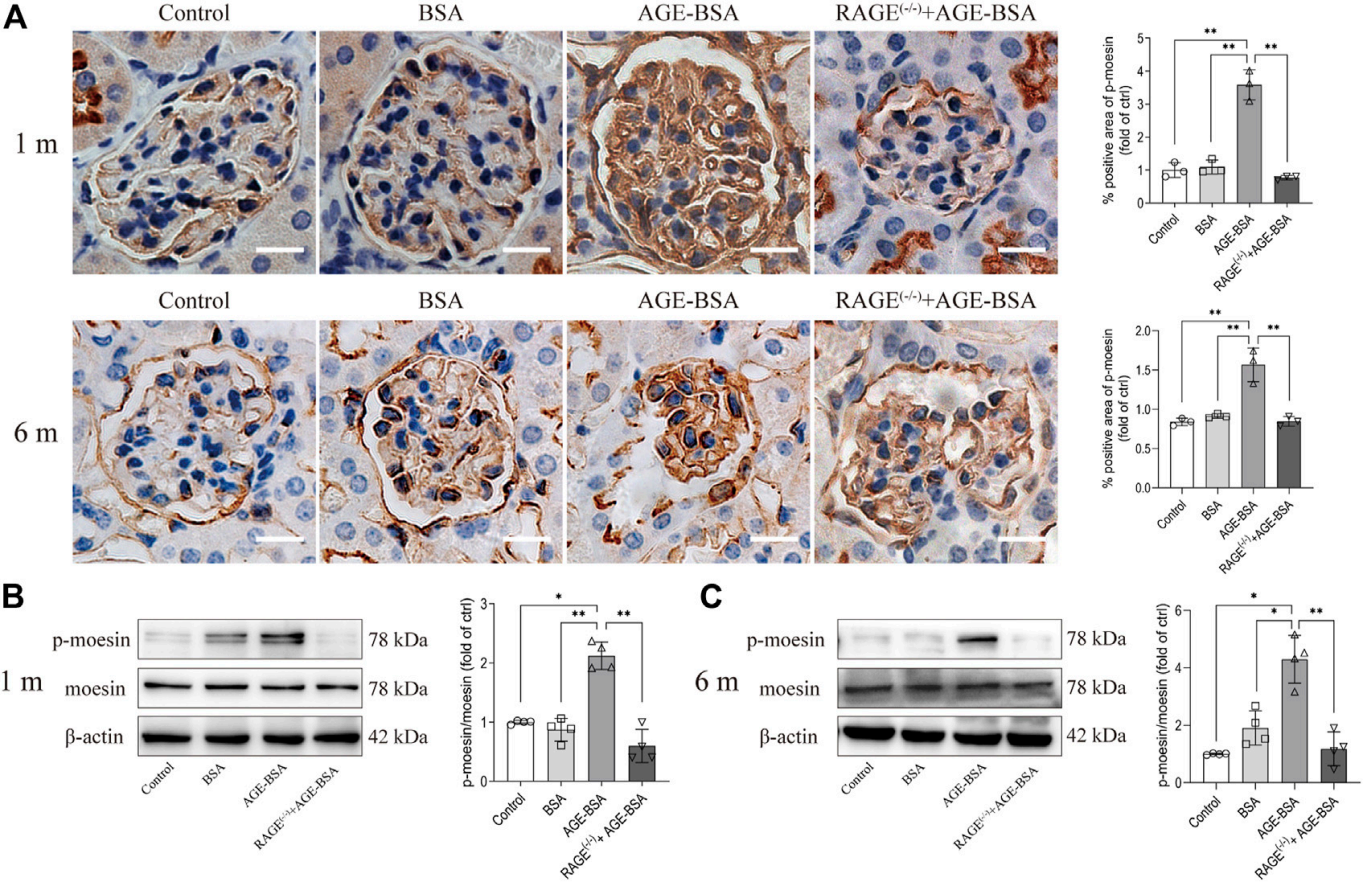
AGE-BSA enhanced the level of moesin phosphorylation in mouse kidneys. **(A)** Immunohistochemical staining of phospho-moesin at 1 and 6 m after treatment. Scale bar = 20 μm. *n* = 3 and **p < 0.01. **(B,C)** Phospho-moesin and total moesin expression in kidneys from AGE-BSA-treated mice for 1 m **(B)** and 6 m **(C)** were detected by immunoblotting. *n* = 4, *p < 0.05, and **p < 0.01.

The immunohistochemical and Western blot assays for moesin phosphorylation were performed in kidneys from AGE-BSA-treated mice. The positive staining of phospho-moesin was remarkably increased in glomerular vascular endothelial cells, as well as in endothelial cells in neovessels around glomeruli at 1 m in AGE-BSA-treated WT mice (Figure 6A, upper panel). The staining of phospho-moesin was still strong at 6 m, and the images of phospho-moesin also illustrated the atrophy of glomeruli in long-term AGE treatment (Figure 6A, lower panel). Western blot data (Figures 6B,C) were consistent with the results of immunohistochemistry. No significant changes in moesin phosphorylation were observed in RAGE^(−/−)^ mice treated with AGE-BSA for 1 m or 6 m.

### Phospho-moesin was co-localized with CD34 in glomeruli from advanced glycation endproduct-treated mice

**FIGURE 7:**
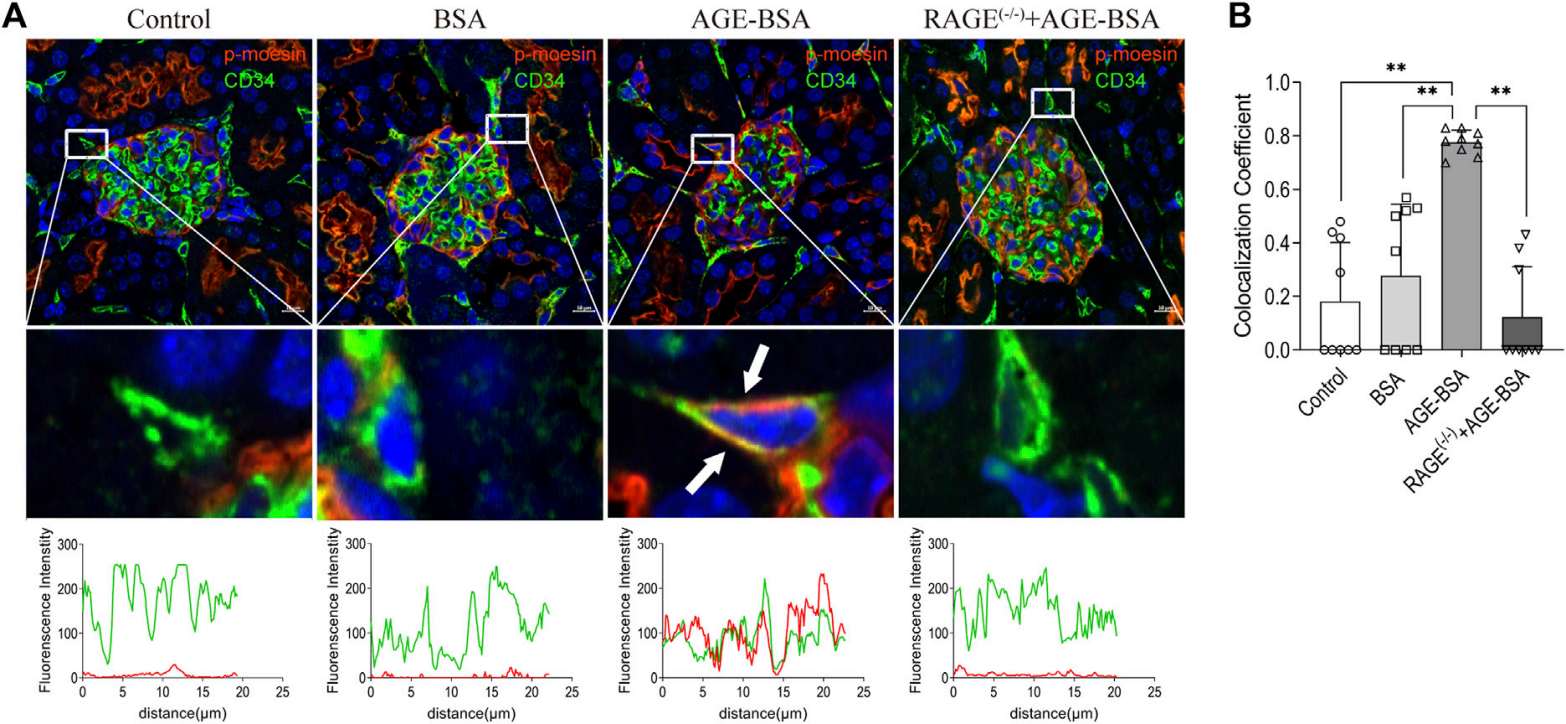
Colocalization of phospho-moesin with CD34 in renal glomeruli under AGE treatment. **(A)** Phospho-moesin (red) and CD34 (green) were presented in renal glomeruli by immunofluorescence assay with TSA (tyramide signal amplification) technology. DAPI (blue) was used to stain nuclei (upper panel). Scale bar = 10 μm. Periglomerular vessel (CD34^+^) was selected for detailed observation (middle panel) of colocalization with phospho-moesin (white arrows). The green and red fluorescence density and localized distance were measured (lower panel). **(B)** Quantification of the fraction of phospho-moesin colocalizing with CD34 in different groups, expressed as the Mander’s overlap colocalization coefficient. *n* = 9 (three different fields analyzed from one sample from three different animals), **p < 0.01.

To elicit the involvement of moesin phosphorylation in AGE-induced renal angiogenesis, we first detected if there was co-localization between phospho-moesin (red) with the endothelial-specific marker CD34 (green). The image shows the orange color in renal glomeruli from AGE-treated mice (Figure 7A). Compared with the control (mean co-localization coefficient: 0.18 ± 0.07, Figure 7B) and BSA-treated (mean co-localization coefficient: 0.28 ± 0.09, Figure 7B) group, the qualitative analysis of fluorescence co-localization reveals more significant phospho-moesin staining (red line) and more merging of red and green in the AGE-BSA-treated group (mean co-localization coefficient: 0.78 ± 0.02, Figure 7B), indicating the increase of phosphorylation of moesin and the co-localization of phospho-moesin with CD34 upon AGE treatment. This result implies that the phosphorylation of moesin happened in endothelial cells and might be important in mediating AGE-induced angiogenesis. No co-localizations were revealed in RAGE^(−/−)^ mice treated with AGE-BSA (mean co-localization coefficient: 0.12 ± 0.06, Figures 7A,B).

### Moesin deficiency attenuated advanced glycation endproduct-induced renal fibrosis

**FIGURE 8:**
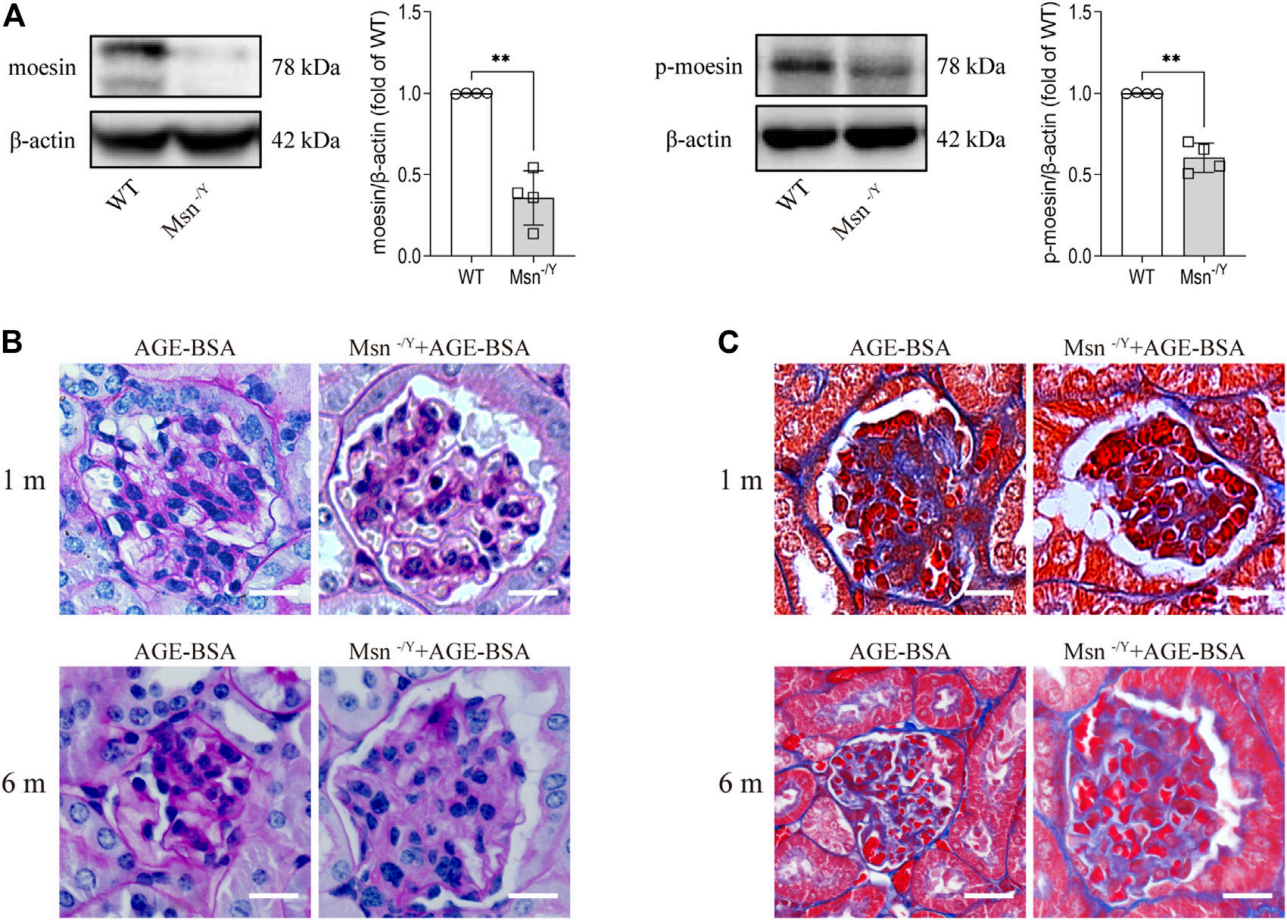
Moesin deficiency attenuated AGE-BSA-induced renal fibrosis. **(A)** Efficiency of moesin knockout in mice was confirmed by Western blot. *n* = 4. **p < 0.01. **(B)** PAS and **(C)** Masson’s staining were conducted in renal tissue from both 1 and 6 m AGE-BSA-treated Msn^−/Y^ mice and compared with AGE-treated WT mice.

To prove that moesin and its phosphorylation play a role in AGE-BSA-induced kidney changes, we applied a moesin-knockout mouse model (Figure 8A) with AGE-BSA treatment. The results revealed that PAS (Figure 8B) and Masson’s trichrome staining (Figure 8C) of renal tissue in AGE-treated Msn^−/Y^ mice were much lighter than that in AGE-treated WT mice, accompanied by improvement of glomerular atrophy, indicating the effect of moesin phosphorylation in AGE-induced renal fibrosis.

### Phosphorylation of moesin T558 is critical in advanced glycation endproduct-induced tube formation of cultured glomerular endothelial cells

**FIGURE 9:**
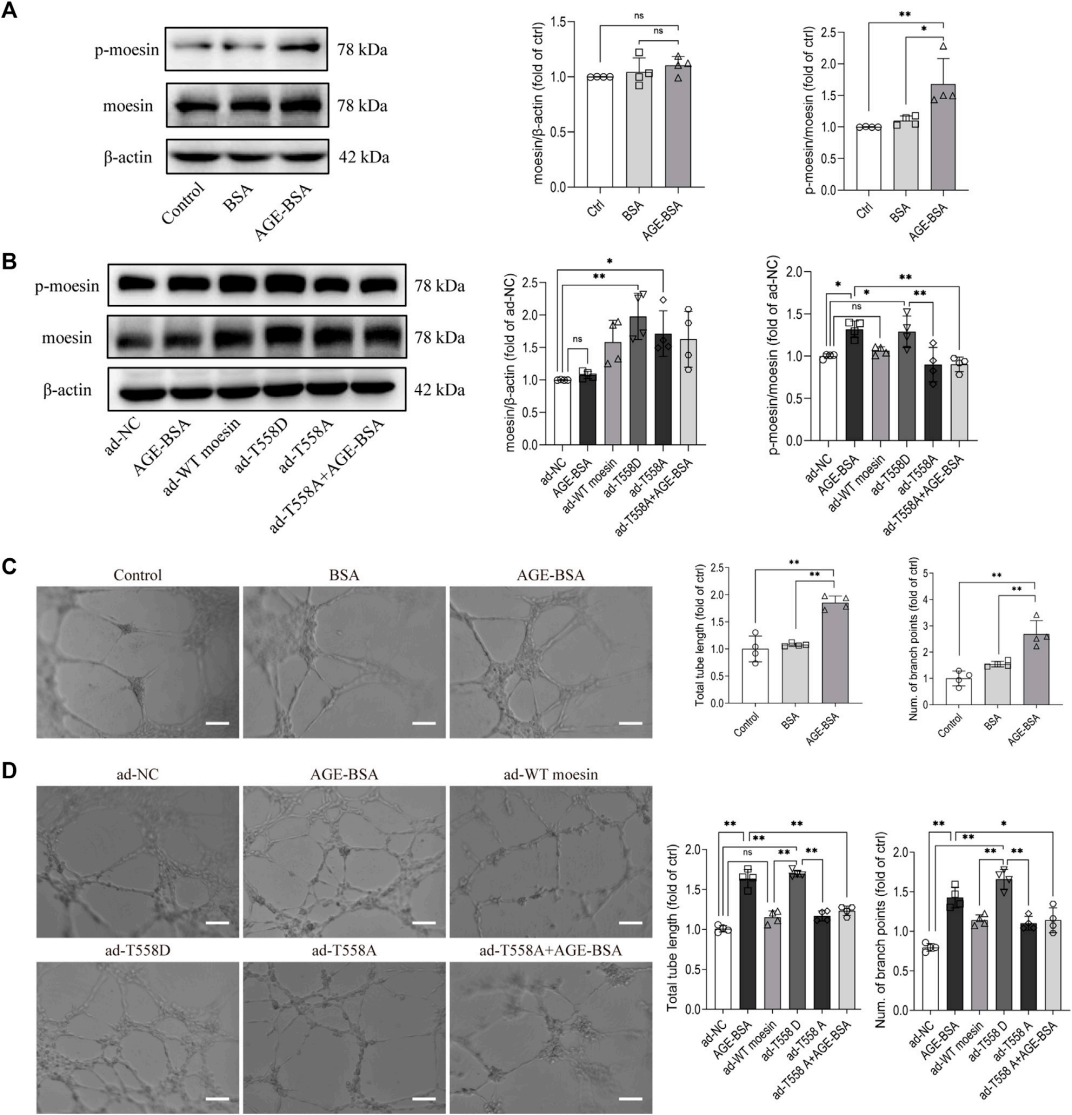
Moesin T558 phosphorylation is important in the AGE-mediated tube formation of GECs. The state of moesin phosphorylation in GECs after AGE-BSA **(A)** and moesin mutant adenovirus **(B)** treatments were detected. Tube formation of GECs treated with AGE-BSA **(C)** or wild-type moesin (ad-WT moesin), moesin T558D (ad-T558D), and moesin T558A (ad-T558A) adenovirus **(D)** is shown. Scale bar = 20 μm. *n* = 4, *p < 0.05, and **p < 0.01.

Our previous studies have suggested the effect of moesin T558 phosphorylation in tube formation in HUVECs (Wang et al., 2016). Herein, we applied cultured GECs to explore if moesin T558 phosphorylation also played a role in AGE-induced tube formation of GECs. Although the efficiency of moesin mutant adenovirus (Figure 9A) and the effect of AGE-BSA on moesin phosphorylation (Figure 9B) in GECs were first confirmed, the results demonstrated that AGE-BSA treatment did significantly enhance GEC tube formation (Figure 9C). The inhibited moesin T558A or activated moesin T558D was applied to GECs, respectively. In comparison with the negative control group, the activating mutant moesin T558D itself could enhance GEC tube formation, while the inhibiting mutant moesin T558A attenuated the tube formation of GECs in quiescent cells, as well as in AGE-BSA-treated cells (Figure 9D). Compared with WT moesin, the activating mutant moesin T558D showed significant enhancement of GEC tube formation, indicating that moesin phosphorylation at T558 has an important role in AGE-induced tube formation, and non-phospho-moesin did not involve in this process (Figure 9D). These results suggested that the phosphorylation of T558 in moesin is important in AGE-induced moesin activation, as well as in AGE-induced glomerular angiogenesis.

## Discussion

This study addressed the direct effects of exogenous AGE-BSA application on the alterations of renal morphology and functions in C57BL/6 male mice. We confirmed that AGE-BSA evokes glomerular angiogenesis at the early stage but glomerular atrophy and renal fibrosis in the advanced stage. From the studies with RAGE^(−/−)^ mice, we concluded that RAGE plays an important role in AGE-BSA-induced chronic renal injury. By applying Msn^−/Y^ mice and interfering with the phosphorylation state of moesin in GECs, we also demonstrated that moesin phosphorylation in T558 was involved in endothelial angiogenesis during the progress of chronic renal injury induced by AGE-BSA.

Primary hyperglycemia triggers endothelial dysfunction, but the endproducts derived from the interaction between excessive glucose and large molecules in plasma and various tissue, namely, AGEs, also play a critical role in the development of diabetic microangiopathy. The accumulation of AGEs in plasma induced prolonged pathological responses, even after hyperglycemia is under control, implying the effect of hyperglycemia memory.

As the most harmful complication of diabetes mellitus, DN has caused wide public concern all over the world. It has been proved that the early onset of abnormal angiogenesis in DN is connected with glomerular hypertrophy and the increased level of albumin in urine, which finally leads to albuminuria and boosts the development and progression of DN (Nakagawa et al., 2009; Nakagawa et al., 2011; Advani and Gilbert, 2012). DN affects almost 35% of patients with diabetes, regardless of their glycemic control, implying the effects of AGEs. This study provides direct evidence that AGEs exert the proangiogenic effect at an early stage by demonstrating the visibly dilated neovessels with specific CD34 staining of proliferative endothelial cells around glomerular capsules.

Podocyte injury is the major cause of proteinuria in chronic kidney disease, and it has been proved that oxidative stress induced by advanced oxidation protein products results in podocyte damage and dysfunction through various signal pathways (Zhang et al., 2017; Zhou et al., 2019). Kim et al. (2018) demonstrated that, in cultured podocytes, AGEs induced disruption of podocyte slit junctions. Our study in this exogenous AGE injection mouse model revealed that AGE application for 1 m could have induced the fusion and detachment of podocyte foot processes with decreasing foot process junctions in glomeruli. These endothelial angiogenesis and podocyte foot process effacement were coincident with the early increase in the albumin level in urine at 1 m after the AGE challenge. The normal and stable morphological and functional situation in blank and BSA control mice supports the idea that AGE accumulation is an independent pathogenic factor in triggering the development of chronic kidney injury.

On the other hand, the present study shows that distinct from the early stage of AGE-BSA treatment, lower levels of angiogenesis and reduced glomerular capillaries were featured in the later period of AGE-BSA treatment, which was postulated to be linked to glomerulosclerosis (Hohenstein et al., 2006; Baelde et al., 2007). Our results also indicated that renal structural disorders and fibrosis were significantly aggravated, but no new vessels were presented around the renal capsule with the time extension of AGE-BSA treatment. The renal dysfunction was demonstrated by an impaired glomerular filtration barrier with an increased ratio of urine albumin-to-creatinine even at the early stage of AGE-BSA treatment. Although these results support the notion that the presence of AGEs exerts an independent effect in promoting fibrosis (Vlassara et al., 1994), the present study unveiled the dynamic changes of vasodilation and angiogenesis and its possible influence on the glomerular filtration barrier during the development of AGE-induced chronic kidney injury.

RAGE is the most considered receptor for AGEs. Interaction of AGEs with RAGE plays a pivotal role in the pathogenesis of diabetic complications (Ahmed, 2005). Renal biopsies from patients with diabetic nephropathy showed that RAGE is expressed in various cell types of the kidney, such as podocytes and glomerular endothelial cells (Tanji et al., 2000). The level of RAGE was significantly enhanced in diabetic mice, and blockage of RAGE prevented glomerulus damage (Wendt et al., 2003). In the present study, we used RAGE^(−/−)^ mice to determine the role of RAGE in AGE-BSA-induced renal injury. We found that AGE-induced angiogenesis and albuminuria at the early stage were both abolished, and fibrosis and renal dysfunction were prevented in RAGE^(−/−)^ mice, supporting that AGEs exert pathological impact on the kidney through RAGE in vivo.

Moesin, a member of the ERM family that possesses the FERM domain at the N-terminal, interacts between the plasma membrane and actin cytoskeleton (McClatchey, 2014). Our previous studies showed moesin phosphorylation is involved in AGE-induced angiogenesis in HUVECs (Wang et al., 2016). We have also demonstrated that AGEs inflicted cerebral microangiopathy through activation of the RhoA/ROCK/moesin signaling pathway (Li et al., 2011). It is also suggested that RAGE is the upstream signal for RhoA/ROCK/moesin activation and subsequent pathological angiogenesis (Chen et al., 2020). These data revealed the active role of moesin phosphorylation under the AGE environment *in vitro* and *in vivo*. Recent research implicated that DN is also associated with a genetic predisposition (Pezzolesi et al., 2009). Genome-wide association studies for type 1 diabetes disclosed both *FERMD3* and *CARS* as novel genes in the pathogenesis of DN (Pezzolesi et al., 2009). Millioni et al. (2008) showed enhanced expression of moesin in fibroblasts from type 1 diabetes mellitus patients with nephropathy. In the present study, immunohistochemistry and Western blot assay showed that the level of moesin phosphorylation on T558 residue was significantly enhanced after AGE-BSA treatment and moesin deficiency attenuated AGE-BSA-induced renal fibrosis and dysfunction, suggesting phosphorylation of moesin participated in AGE-induced chronic kidney injury. In addition, the involvement of phospho-moesin in AGE-induced renal angiogenesis was further confirmed by the co-localization of phosphor-moesin and CD34 around the glomerulus in the kidney from AGE-BSA-treated mice, as well as the angiogenetic effect of moesin T558D on tube formation of GECs. There have been studies showing that moesin phosphorylation might also be involved in the process of fibrosis in different organs (Okayama et al., 2008; Chen et al., 2014; Zhu et al., 2014), indicating moesin phosphorylation exerts sustained effect in the progress of AGE-induced kidney injury.

One of the limitations to this study is that although the results indicated that AGEs mediate glomerular angiogenesis at the early stage but glomerular atrophy and renal fibrosis at the late stage, it lacks a more in-depth exploration of mechanism based on molecular biology. It has been reported that angiogenic growth factor expression is related to capillary growth in early diabetes or rarefaction in late diabetes (Advani and Gilbert, 2012). Our unpublished data preliminarily indicate that AGE-BSA is also involved in the regulation of vascular endothelial growth factor and its receptor. Another limitation to this study is that the role of moesin phosphorylation in AGE-induced chronic kidney injury with characteristic patterns in different stages was yet to be explored. Although there have been reports suggesting the effects of moesin phosphorylation both in the angiogenesis and in fibrosis of different organs, it is still unclear whether p-moesin exerted different effects at different stages. A lot more work needs to be conducted in the future.

In conclusion, this study provides evidence of dynamic pathological changes in the kidney under sustained stimulation of AGE-BSA *in vivo*. We conclude that AGEs mediate morphological disorders and dysfunctions of the kidney through RAGE. Phosphorylation of moesin is involved in this progress, but the role and molecular mechanism of phospho-moesin in AGE-induced renal malfunction still need further exploration.

## Data Availability

The original contributions presented in the study are included in the article/Supplementary Material; further inquiries can be directed to the corresponding authors.

## Supplementary material

SUPPLEMENTARY FIGURE S1 Expression of CD34 and the content of Col-I were not changed in the kidney tissue from AGE-BSA-treated mice. The expression of CD34 **(A)**and the content of Col-I **(B)** in the kidney tissue from AGE-BSA-treated mice for 1 m and 6 m were detected by immunoblotting. *n*=4; ns, no significance.

SUPPLEMENTARY FIGURE S2 The efficiency of RAGE knockout in mice. **(A)** Genotyping was conducted using mouse tail tissue with primers of 5′-CCA​GAG​TGA​CAA​CAG​AGC​AGA​C-3′ (primer1); 5′-CCT​CGC​CTG​TTA​GTT​GCC​CGA​C -3′ (primer 2) and 5′-GGT​CAG​AAC​ATC​ACA​GCC​CGG​A -3′ (primer 3), respectively. **(B)** RAGE expression was detected by immunoblotting in kidney tissue from WT and RAGE^(−/−)^ mice. *n* = 4, **p*<0.05.
